# Papillary Meningioma: Case Presentation with Emphasis on Surgical and Medical Therapy of a Rare Variant of Meningioma

**DOI:** 10.3390/diseases9030063

**Published:** 2021-09-17

**Authors:** Gerardo Cazzato, Valeria Internò, Antonietta Cimmino, Raffaella Messina, Marco Tucci, Teresa Lettini, Leonardo Resta, Giuseppe Ingravallo

**Affiliations:** 1 Section of Pathology, Department of Emergency and Organ Transplantation (DETO), University of Bari Aldo Moro, 70124 Bari, Italy; micasucci@inwind.it (A.C.); lettinit@yahoo.com (T.L.); leonardo.resta@uniba.it (L.R.); 2 Department of Biomedical Sciences and Human Oncology, University of Bari Aldo Moro, 70124 Bari, Italy; valeria.interno@libero.it (V.I.); marco.tucci@uniba.it (M.T.); 3 Neurosurgery Unit, Department of Basic Medical Science, Neurosciences and Sense Organs, “Aldo Moro” University of Bari Medical School, 70124 Bari, Italy; raffaella.messina@uniba.it

**Keywords:** papillary meningioma, neurosurgery, oncology, rare tumours

## Abstract

Meningioma is one of the most frequent neoplasms of all in the central nervous system. Different variants are known, and of these some have peculiar characteristics, both from a morphological point of view and from a biological point of view. Here, we present a rare case of relapsed papillary meningioma in a young patient, focusing on histological characteristics, medical-surgical therapy and focusing on the risk of progression and/or recurrence of the lesion if not completely eradicated. Finally, we provide detailed molecular characteristics of the case in question.

Meningiomas represent a group of slow-growing neoplasms that arise from the arachnoidal cap cells of the meninges [1,2]. The majority of meningiomas histologically correspond to grade I tumours, according to the 2016 World Health Organization (WHO) classification of the central nervous system [2]. In the literature, there are various histopathological variants of meningiomas, each one with a peculiar biological behaviour and, consequently, with a different clinical outcome [2,3]. Meningiomas are most frequently diagnosed in women and usually express progesterone receptors [1,3]. Among the different histopathological variants, papillary meningioma (PM) constitutes <1% of all meningiomas and is classified as a rare variant, histologically corresponding to WHO grade III, known as malignant meningiomas [1,2,3,4,5,6,7,8,9,10,11].

Here, we present a case of papillary meningioma that arose in a 42-year-old woman and relapsed seven years after the first surgical removal and adjuvant radiation therapy. We then explored the available systemic strategies available in the existing literature.

In 2010, a 42-year-old woman reported a headache, speech difficulties and hypersomnia for about one week. After careful neurological consultation and the persistence of symptoms, we opted to perform a functional magnetic resonance imaging (MRI) with gadolinium, which revealed the presence of a neoplastic lesion in the left frontoparietal area. The patient underwent removal of the lesion (Simpson’s grade 2 the boundary of the tumour was unclear and invaded the surrounding brain tissue) and the histopathological analysis revealed a PM. Then, due to the histopathological analysis and the residual tumour, a conformational radiotherapy adjuvant treatment was performed (60 Gy per fraction of 2 Gy in 30 sessions). The disease dd not reoccur until 2015, when a brain MRI with gadolinium documented disease recurrence in the same area of the previous surgical removal. The patient, again, underwent the neurosurgical removal of the lesion. She then decided to disregard the radiological follow-up.

In November 2020, she reported again headache, sleep disturbances and functional symptoms. A brain MRI was performed (Figure 1A,B) and showed a giant meningioma in the left parietal area of 5 × 5 cm. The mass was surrounded by a perifocal oedema with consecutive compression and dislocation to the right of the ventricular system. The temporal horn of the ipsilateral ventricle resulted in communication with the subarachnoid space. The administration of the gadolinium determined inhomogeneous accentuation of the mass. The patient was again subjected to tumour excision, which was performed at the Neurosurgery department of the University Hospital of Bari. Table 1 summarizes the main steps of the patient.

The lesion was fixed in 10% buffered formaldehyde and sent to our pathology laboratory. After sampling, processing, paraffin embedding and microtome cutting, five-micron thick sections were obtained and stained with Haematoxylin Eosin for observation under an optical microscope. The presence of a proliferation, characterized by pseudopapillary architecture with loss of cellular cohesion, clinging of tumour cells in blood vessels and the presence of nucleus-free perivascular zones that resembled the ependymoma pseudo-rosette was demonstrated (Figure 2A–C). Immunohistochemically, there was a strong expression of Epithelial Membrane Antigen (EMA, Figure 2D) and Vimentin (not shown), while the progesterone receptor (PR) was focally positive (<10%), as is usual in anaplastic meningiomas that do not usually have a positive expression of PR. The neoplastic proliferation fraction (Ki67+) was >20%. In consideration of the histological and immunophenotypic picture, the diagnosis of papillary meningioma, grade III (WHO) was made. The patient then refused any other type of adjuvant treatment and was lost at follow-up.

From a molecular point of view, cytogenetic investigations were carried out, which revealed very interesting and suggestive results; in particular, a characteristic codeletion of chromosomes 1p and 14q was found, the realization of which has recently been suggested as a potential adverse prognostic risk factor in meningioma [12]. There were no NF2 mutations [13], but there were two copy number losses of the PBRM1 gene [14]. There were no other mutations in other genes, such as BAP1 or others.

Regarding therapeutic systemic strategies for recurrent PM, a rare but aggressive variant as demonstrated in this case report, there is a lack of universally accepted guidelines after re-surgery and re-irradiation therapy [15]. Regarding prognostic factors, the impact of the extent of surgical resection is well-known, otherwise, postoperative radiation therapy on survival of PM patients has not been well studied due to the limited number of cases of PM. However, Simpson grading prognostic score is accepted and individuates four different subgroups (grade I to IV) based on residual tumour or infiltrated dura. In particular, residual tumour tissue after surgery or infiltrated dura result in a significant risk of tumour recurrence. Moreover, it remains unclear if the prognostic role of Simpson grading stands nowadays, especially with regard to the adjuvant radiotherapy for selected cases of subtotal resections (Simpson grade IV) that are associated with higher risk for tumour recurrence [16]. Furthermore, Simpson grade I–III (no or minimal residual disease) vs. grade ≥IV (macroscopic residual disease) resections allow a more exact prediction of the risk of postoperative tumour relapse [17]. A recent published analysis of 108 patients with papillary meningioma identifies EOR as an important prognostic factor, even in multivariate analysis. To date, patients who underwent gross total resection (GTR) had a significantly higher probability of disease-specific survival compared with those who underwent subtotal resection (STR)**.** Concerning the presumed prognostic significance of the progesterone receptor expression detectable by immunohistochemistry, a recent work by Hua L. et al. [18] did not identify a relationship between expression and patient outcome, unlike the immunoexpression of the oestrogen receptor.

Regarding post-operative management, adjuvant radiotherapy is associated with DSS in univariate analysis. As a result, GTR resulted in a 5-year PFS rate of 36.7% compared with 0% in the case of STR. Sumner et al. explored the survival benefit of postoperative radiation therapy in PM and demonstrated improved survival, arguing for postoperative radiation therapy to manage those aggressive tumours [19]. As a result, despite the lack of prospective studies, postoperative radiotherapy represents the standard of care. Another possible option for post-operative treatment is represented by the proton therapy. This type of radiotherapy allows the total dose to be increased and provides the greatest coverage of the target volume, while not being limited by the large size or abnormal shape of the target tumour.

Regarding systemic adjuvant strategies, chemotherapy has failed in controlling malignant meningioma growth and has only been investigated in small clinical trials given the paucity of this tumour type. Established chemotherapeutic agents in the treatment of other systemic tumours have not replicated their robust activity in controlling malignant meningioma growth. In particular, hydroxyurea, an oral ribonucleotide reductase inhibitor, halts meningioma cell growth, the S-phase of the cell cycle, and induces apoptosis. One preliminary report found that hydroxyurea prevented recurrent growth for 24 months in a patient following GTR of a malignant meningioma [20]. In addition, a prospective study of 16 patients with recurrent meningiomas, five of whom were classified as grade III, studied the effect of a somatostatin analogue on radiographic response and progression-free survival at six months. These tumours were shown to overexpress somatostatin receptors by octreotide scintigraphy and so were considered responsive to a long-acting somatostatin analogue. Only one of the five Grade III meningiomas demonstrated a partial response to the treatment, while another Grade III patient showed stable disease with the remaining three showing progressive disease [19,20]. In the case of recurrent malignant meningiomas, there is a less codified approach. The early consensus is repeat surgery, but the role of adjuvant therapies such as radiation and chemotherapy are less convincing [15].

Papillary meningioma is a rare variant of meningioma, defined by the presence of a perivascular pseudopapillary pattern constituting most of the tumour [1,5], and it is considered as WHO grade III (malignant meningioma) [1]. Histologically, pseudo-papillary architecture is characterized by loss of cohesion, with the presence of pseudo-rosettes resembling ependymoma [1,2] with occasional foci of necrosis. Immunostaining for EMA and Vimentin are, frequently, strongly positive, with different and wide expressions of Progesterone Receptor and Ki67+ which is usually around 5–6% [4,5]. Some meningiomas combine a papillary architecture with rhabdoid cytology and this finding was correlated with an even more aggressive clinical-biological behaviour [6,7]. Generally, papillary meningiomas have an invasive tendency, including brain invasion [1,6], recurrence and metastasis [1,7,8]. It is important to underline that other meningioma subtypes can also exhibit a papillary pattern, but that the histological diagnosis of PM should only be made when almost the entire mass has this type of histological pattern [1,7]. Furthermore, given the high incidence of being faced with the possibility of an impromptu, it can be very difficult to discriminate this type of meningioma from high-grade glial lesions [9]. Recently, some authors as Cheng and Al. described intra-tumoral cystic changes in PM without correlating with the increased aggression of biological behaviours [10] and, above all, once recurred and re-irradiated, no systemic treatment strategies are known to be efficacious in controlling the tumour growth.

Finally, we have reported our experience with a rare case of papillary meningioma that has recurred twice, and we discussed the various steps for a correct medical-surgical management of this entity. Future directions will go towards a greater understanding of the etiopathogenesis of the lesion and, possibly, the development of drugs able to reduce the recurrence of this aggressive subtype of meningioma.

## Figures and Tables

**Figure 1:**
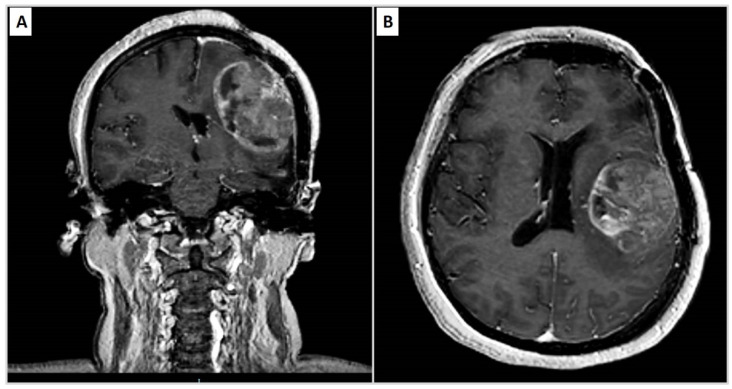

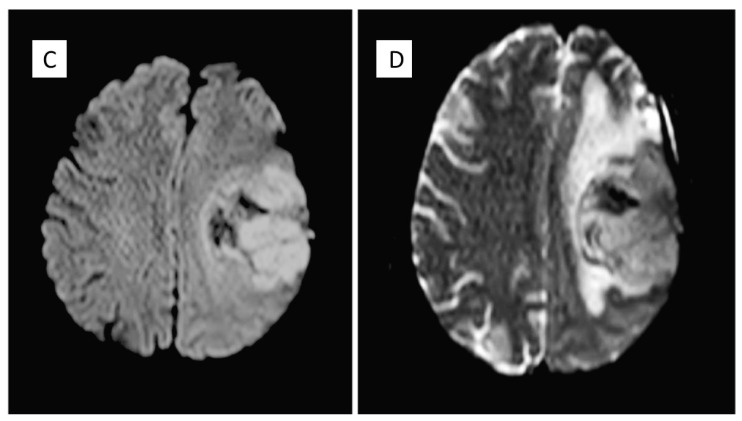
(**A**,**B**) Axial and coronal projections. Large meningioma in the left parietal area of 5 × 5 cm. The mass is surrounded by perifocal oedema with consecutive compression and dislocation to the right of the ventricular system. The temporal horn of the ipsilateral ventricle results in communication with the subarachnoid space. The administration of the gadolinium determines inhomogeneous accentuation of the mass. (**C**,**D**) PM displays an heterogenous hyperintensity in DWI surrounded by an hypointense oedema.

**Figure 2:**
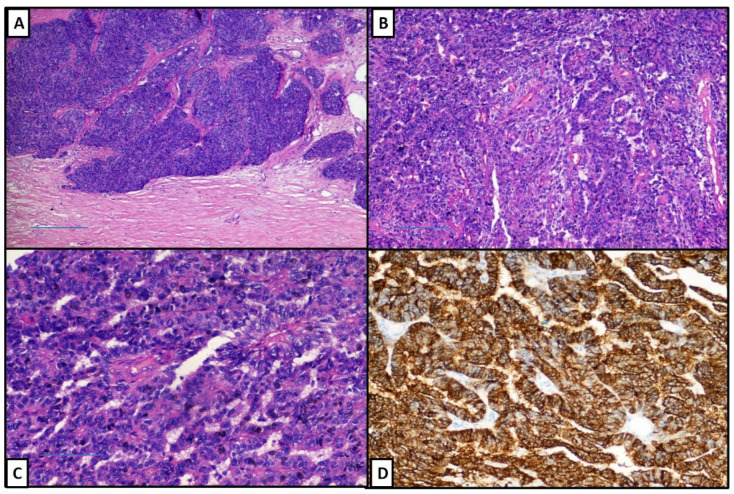
(**A**–**C**) The presence of a proliferation characterized by pseudopapillary architecture, with loss of cellular cohesion, clinging of tumour cells in blood vessels and the presence of nucleus-free perivascular zones that resembled the ependymoma pseudo-rosette was demonstrated. (Haematoxylin-Eosin, Original Magnification: 10×, 20× and 40×, respectively). (**D**) Immunohistochemically, there was a strong expression of Epithelial Membrane Antigen (EMA, immunohistochemistry, original magnification: 20×).

**Table 1:** Summary of the patient’s medical history mentioning symptoms, diagnosis, and therapeutic treatment.

Years	Symptoms	Diagnosis	Treatment
2010	Headache, speech difficulties and hypersomnia	Neoplastic lesion in the left frontparietal area compatible with papillary meningioma	Surgical removal and radiotherapy adjuvant treatment
2015	Headache	Recurrence of PM	Neurosurgical removal
2020	Headache, sleep disturbances and functional symptoms	Recurrence of PM	Tumour excision

## Data Availability

Not applicable.
